
JOURNAL INFORMATION
==============================
NLM Title Abbreviation: Cell Prolif
Journal ID: Cell Prolif
NLM Journal ID: 9105195
Journal ID: CPR

Cell Proliferation

ISSN: 0960-7722
EISSN: 1365-2184
Publisher: Wiley

ARTICLE INFORMATION
==============================
PMCID: PMC11474677
DOI: 10.1111/cpr.13268
Article ID: CPR13268
Article version: 1
Subjects: Issue Information, Issue Information

Issue Information

Electronic publication date: 2023 Nov 3
Publication date: 2023 Nov
Volume: 56
Issue: 11
Electronic Location ID: e13268
Copyright: © 2023 The Authors. Cell Proliferation published by Beijing Institute for Stem Cell and Regenerative Medicine and John Wiley & Sons Ltd.
License: This is an open access article under the terms of the http://creativecommons.org/licenses/by/4.0/ License, which permits use, distribution and reproduction in any medium, provided the original work is properly cited.
License URL: https://creativecommons.org/licenses/by/4.0/

Figure count: 0
Table count: 0
Page count: 2

source-schema-version-number: 2.0
cover-date: November 2023
details-of-publishers-convertor: Converter:WILEY_ML3GV2_TO_JATSPMC version:6.4.9 mode:remove_FC converted:15.10.2024

==============================

Cell
Proliferation

**Editor-in-Chief**
**Professor Q. Zhou**
Institute of Zoology
Chinese Academy of Sciences
1 Beichen West Road, Chaoyang District
Beijing 100101
China

**Deptuy Editor**
**Prof. Y. Lin**
Sichuan University
No 14, 3rd Sec, Renmin South Road
Chengdu 610041
China

**Associate Editors**
**Dr. L. Wu**
InVitro Cell Research, LLC
Englewood, NJ
USA

**Prof. N. Shyh-Chang**
Chinese Academy of Sciences,
Beijing, China

**Prof. G. Stacey**
University of Suffolk,
UK

**Editorial Board**
M. R. Alison, Royal London Hospital, London, UK
C. Booth, Epistem, Manchester, UK
X. Cai, Sichuan University, Chengdu, China
J. Costa-Rodrigues, University of Porto, Porto, Portugal
M. G. Daidone, Instituto Nazionale per lo Studio, e la Cura dei Tumori, Milan, Italy
Z. Darzynkiewicz, University of New York, New York, USA
M. Eastwood, University of Westminster, London, UK
W. Engstrom, University of Uppsala, Uppsala, Sweden
R. Gottardi, University of Pittsburgh, Pittsburgh, USA
J. Hua, Northwest A&F University, Yangling, China
J. Jiao, Institute of Zoology, CAS, China
A. Khademhosseini, Brigham and Women's Hospital, Harvard University, Boston, USA
J. Kovar, Charles University, Prague, Czech Republic
D. R. Lee, CHA University, Seoul, Republic of Korea
L. Li, University of Kansas Medical Center, Kansas, USA
W. Li, Institue of Zoology, CAS, China
G. Liu, Insitute of Biophysics, CAS, China
C. McGuckin, Conoworld, France
H. Mehmet, Merck Ltd, USA
C. Nadalutti, University of North Carolina, North Carolina, USA
S. C. Ng, Genome Institute of Singapore, Singapore
W. R. Otto, Kingston, UK
M. Pignatelli, University of Glasgow, Glasgow, UK
R. Poulsom, Queen Mary, University of London, London, UK
J. Qu, Institute of Zoology, CAS, China
H. Song, John Hopkins, USA
A. Tàrnok, Klinik fűr KinderKardiologie, University of Leipzig, Leipzig, Germany
S. Totey, Kansiak Research, Mumbai, India
H. Wang, Institute of Zoology, CAS, China
L. Wang, Beijing University of Posts and Telecommunications, Beijing, China
N. A. Wright, Queen Mary, University of London, London, UK
M. C. Yoder, Indiana University School of Medicine, Indianapolis, USA
B. Zhivotovsky, Karolinska Institutet, Stockholm, Sweden

**Aims and Scope**
*Cell Proliferation* is a journal devoted to studies into all aspects of cell proliferation and differentiation in normal and abnormal states; stem cells, cancer, regenerative medicine, cell death; control systems and mechanisms operating at inter-and intracellular, molecular and genetic levels; modification by and interactions with chemical and physical agents; mathematical modelling; and the development of new techniques. In addition to original research papers *Cell Proliferation* publishes invited review articles, book reviews and letters commenting on previously published papers and/or topics of general interest.

**Open Access and Copyright**
All articles published by *Cell Proliferation* submitted after January 2019 are published under the terms of a Creative Commons license.

Copyright on any research article published by *Cell Proliferation* is retained by the author(s).

Further information about open access licenses and copyright can be found at: https://authorservices.wiley.com/open-research/open-access/index.html. Information on the licenses used by *Cell Proliferation* can be found at: https://onlinelibrary.wiley.com/journal/13652184

**Disclaimer**
The Publisher, Beijing Institute for Stem Cell and Regenerative Medicine and Editors cannot be held responsible for errors or any consequences arising from the use of information contained in this journal; the views and opinions expressed do not necessarily reflect those of the Publisher, Beijing Institute for Stem Cell and Regenerative Medicine and Editors, neither does the publication of advertisements constitute any endorsement by the Publisher, Beijing Institute for Stem Cell and Regenerative Medicine and Editors of the products advertised.

Wiley Open Access articles posted to repositories or websites are without warranty from Wiley of any kind, either express or implied, including, but not limited to, warranties of merchantability, fitness for a particular purpose, or non-infringement. To the fullest extent permitted by law Wiley disclaims all liability for any loss or damage arising out of, or in connection, with the use of or inability to use the content.

**Note to NIH grantees**
Pursuant to NIH mandate, Wiley will post the accepted version of contributions authored by NIH grant-holders to PubMed Central upon acceptance. This accepted version will be made publicly available 12 months after publication. For further information, see www.wiley.com/go/nihmandate

**Production editor**
Maricris Apostol (Email: cpr@wiley.com)

For submission instructions, subscription and all other information view this journal online at https://onlinelibrary.wiley.com/journal/13652184
